# The role of moesin in diagnosing and assessing severity of lymphangioleiomyomatosis

**DOI:** 10.1186/s12931-024-02685-6

**Published:** 2024-01-25

**Authors:** Xixi Song, Hui Cai, Wenjun Peng, Ke Chen, Zilinuer Abuduxukuer, Yingying Zeng, Guiping Zhu, Chong Lu, Yu Chen, Jian Wang, Ling Ye, Meiling Jin

**Affiliations:** 1 Department of Allergy, Zhongshan Hospital, Fudan University, Shanghai, China; 2 Department of Pulmonary and Critical Care Medicine, Zhongshan Hospital, Fudan University, Shanghai, China

**Keywords:** Lymphangioleiomyomatosis, Biomarker, Proteomics, Moesin, VEGF-D, Sirolimus

## Abstract

**Background:**

Lymphangioleiomyomatosis (LAM) is a rare disease which is easily misdiagnosed. Vascular endothelial growth factor D (VEGF-D), as the most common biomarker, however, is not so perfect for the diagnosis and severity assessment of LAM.

**Materials and methods:**

The isobaric tags for relative and absolute quantitation (iTRAQ)-based method was used to identify a cytoskeleton protein, moesin. 84 patients with LAM, 44 patients with other cystic lung diseases (OCLDs), and 37 healthy control subjects were recruited for collecting blood samples and clinical data. The levels of moesin in serum were evaluated by ELISA. The relationships of moesin with lymphatic involvement, lung function, and treatment decision were explored in patients with LAM.

**Results:**

The candidate protein moesin was identified by the proteomics-based bioinformatic analysis. The serum levels of moesin were higher in patients with LAM [219.0 (118.7–260.5) pg/mL] than in patients with OCLDs (125.8 ± 59.9 pg/mL, *P* < 0.0001) and healthy women [49.6 (35.5–78.9) ng/mL, *P* < 0.0001]. Moesin had an area under the receiver operator characteristic curve (AUC) of 0.929 for predicting LAM diagnosis compared to healthy women (sensitivity 81.0%, specificity 94.6%). The combination of moesin and VEGF-D made a better prediction in differentiating LAM from OCLDs than moesin or VEGF-D alone. Moreover, elevated levels of moesin were related to lymphatic involvement in patients with LAM. Moesin was found negatively correlated with FEV_1_%pred, FEV_1_/FVC, and DLCO%pred (*P* = 0.0181, *r* = − 0.3398; *P* = 0.0067, *r* = − 0.3863; *P* = 0.0010, *r* = − 0.4744). A composite score combining moesin and VEGF-D improved prediction for sirolimus treatment, compared with each biomarker alone.

**Conclusion:**

Higher levels of moesin in serum may indicate impaired lung function and lymphatic involvement in patients with LAM, suggest a more serious condition, and provide clinical guidance for sirolimus treatment.

## Introduction

Lymphangioleiomyomatosis (LAM) is a low-grade neoplasm characterized by cystic lung destruction, renal angiomyolipomas (AMLs), abdominal lymphangioleiomyomas, and chylous fluid accumulations [1]. It is a rare, chronic progressive disease primarily affecting reproductive-age women when tuberous sclerosis complex 1/2 (TSC1/2) genes mutation occurs in germline or somatic cells, which separately refers to tuberous sclerosis complex associated LAM (TSC-LAM) or sporadic LAM (S-LAM) [2]. Mutation in either the TSC1 or TSC2 gene, mostly the loss of heterozygosity of TSC2, results in the hyperactivation of mammalian target of rapamycin complex 1 (mTORC1), which increases the proliferation and growth of LAM cells [2, 3]. The pathological characteristics of LAM include multiple air-filled cystic spaces and abnormal proliferation of smooth muscle-like cells (LAM cells) around terminal bronchioles, lymphatic vessels, and small blood vessels. Immunohistochemical staining reveals positivity for smooth muscle antibodies (SMA) and human melanoma black-45 (HMB-45), and estrogen and progesterone receptors may also be positive [4, 5]. These pulmonary pathological changes contribute to the progressive worsening of respiratory symptoms, such as dyspnea following exertion, recurrent spontaneous pneumothorax, refractory chylothorax, hemoptysis, and ultimately respiratory failure [6]. Sirolimus is a first-line medication currently used for the treatment of LAM. It is an effective inhibitor of mTORC1, whose safety and efficacy have been demonstrated in treating LAM by several clinical trials [7–9].

Due to its rare nature, LAM is difficult to diagnose, particularly in patients with atypical manifestations. Serum vascular endothelial growth factors D (VEGF-D) is a noninvasive biomarker, whose concentration over 800 pg/mL helps confirm LAM diagnosis in women with compatible clinical characteristics and cystic change on high- resolution computed tomography (HRCT) of the chest [5]. VEGF-D is a ligand for the lymphatic growth factor receptor 2 and vascular endothelial growth factor receptor 3 that induces formation of lymphatics and promotes the spread of tumor cells [10]. It is also reported that a high VEGF-D level predicts an involvement of lymphatic system and connects with the severity and progression of LAM [11–13]. However, normal VEGF-D concentration does not exclude LAM diagnosis [10]. And it remains controversial about the correlation between VEGF-D reduction and the effective response of sirolimus on lung function [11, 14]. Therefore, further research encourages the discovery of new biomarkers that can improve the diagnosis, assessment of disease severity and prognosis of LAM.

Recently, the application of new technologies has expanded the diversity of potential biomarkers. A study applied a cross-disease approach that analyzed gene expression profiles of breast cancer and used liquid chromatography-mass spectrometry (LC–MS/MS) to quantify seven metabolites in plasma samples from patients with LAM and healthy women [15]. Four metabolites were found to be abundant in LAM samples, with 1-methylimidazole-4-acetic acid being more abundant in LAM plasma than any other group [15]. Another study also used a mass spectrometry proteomics approach to identify vitamin D binding protein as a new biomarker for assessing disease severity and clinical outcome in patients with LAM [16].

The studies above used mass spectrometry (MS)-based proteomics and metabolomics to study protein characteristics on a large scale to search for sensitive indicators for disease diagnosis or prognosis. Isobaric tags for relative and absolute quantitation (iTRAQ), one of the new high-throughput proteomics technologies, is used for quantitative research and has been extensively applied in oncology research [17]. In 2014, Nessa Banville et al. used an iTRAQ-based approach in the serum of patients with LAM to identify a significantly altered protein network which was connected to cell trafficking and extracellular matrix remodeling [18]^.^ However, their study didn’t further explore the clinical significance or mechanism of the protein network. Here, we employed the iTRAQ-based method and bioinformatic analysis to identify serum differentially expressed proteins (DEPs) linked with cell invasion and metastasis in LAM. It was found that moesin, belonging to Ezrin-Radixin-Moesin (ERM) family that was related to cell metastasis, was increased in serum of patients with LAM. Additionally, we investigated the potential of moesin as a diagnostic biomarker and its ability to predict disease severity and sirolimus treatment in patients with LAM.

## Materials and methods

### Subjects and clinical data collection

**Fig. 1:**
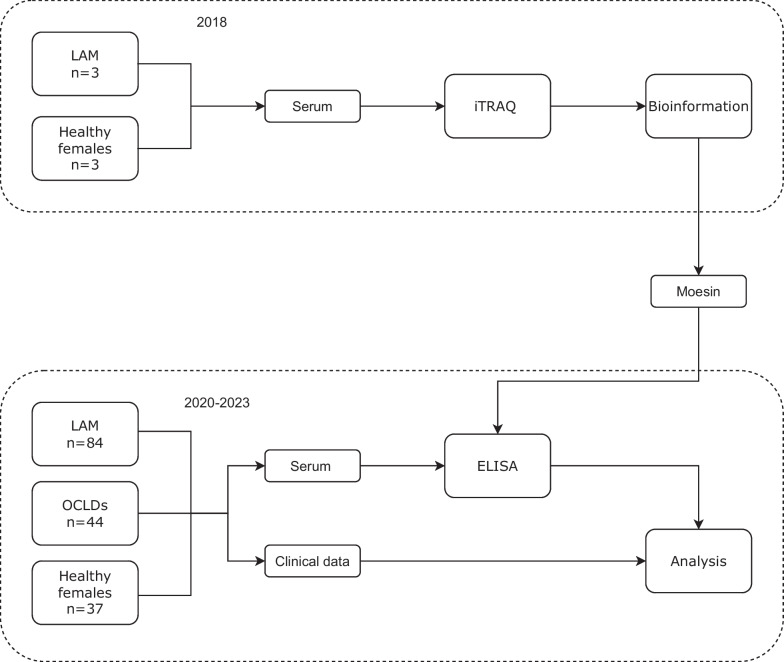
The workflow of the study. The initial six individuals were not included in the validation cohort. *LAM* Lymphangioleiomyomatosis; *iTRAQ* Isobaric tags for relative and absolute quantitation; *OCLDs* Other cystic lung diseases; *ELISA* Enzyme-linked immunosorbent assay

All the participants in our study were recruited from Department of Pulmonary and Critical Care Medicine, Zhongshan Hospital, Fudan University from 2018 to 2023. Baseline clinical characteristics such as age, history of pneumothorax, accumulation of chyle, presence of tuberous sclerosis, AMLs, lymphangioleiomyomas, pulmonary function tests, lung CT scans, and treatment, were recorded through electronic clinical records or telephone follow-ups. All subjects provided written informed consent. The peripheral blood samples of these participants were collected. After centrifugation at 4000 rpm for 10 min, serum samples were obtained and stored at − 80 ℃ for further analysis. The workflow of this study was illustrated in Fig. 1.

The diagnosis of LAM was made by qualified pulmonary physicians according to the guidelines of the American Thoracic Society/Japanese Respiratory Society [5]. The other cystic lung diseases (OCLDs) group included patients with Sjögren’s syndrome (SS), ANCA-associated vasculitis (AAV), systemic lupus erythematosus (SLE), familial vesicular disease, etc. Sirolimus therapy was initiated in LAM patients if they met at least one of the following criteria: (1) FEV_1_%pred < 70% or an annual loss of FEV_1_ ≥ 90 mL/year, (2) symptomatic chylous fluid accumulations [19], (3) an AML or lymphangioleiomyomas ≥ 4 cm in diameter /multiple AMLs or lymphangioleiomyomas, and (4) TSC-LAM [20]. Patients failing to meet the aforementioned criteria did not receive sirolimus therapy and were subject to close observation.

The iTRAQ-based bioinformatic analysis was performed on serum samples from three S-LAM patients and matched healthy women (the discovery cohort) to identify DEPs. One of the three LAM cases with diffuse pulmonary cystic changes alone was diagnosed based on the transbronchial lung biopsy, and the other two were diagnosed on the characteristic pulmonary changes plus renal AMLs. None of these had a history of pneumothorax, chylothorax, chylous ascites, or lymphangioleiomyomas (Table 1). The validation cohort, including 84 patients with LAM, 44 patients with OCLDs, and 37 healthy women, were selected to further verify candidate DEPs in LAM. The initial six individuals were not included in the validation cohort.

**Table 1:** Clinical features of LAM patients selected for iTRAQ detection

	P1	P2	P3
Age, y	29	28	30
Pneumothorax	N	N	N
Chylothorax or chylous ascites	N	N	N
Renal AMLs	Y	Y	N
Lymphangioleiomyomas	N	N	N
VEGF-D, pg/mL	3039.34	1306.10	1933.01
Pathology reports	Epithelioid angiomyolipoma (Right nephrectomy)	NA	Consistent with LAM (TBLB)

*iTRAQ* Isobaric tags for relative and absolute quantitation; *N* No; *Y* Yes; *NA* not applicable; *AMLs* angiomyolipomas; *VEGF-D* vascular endothelial growth factor D; *TBLB* Transbronchial lung biopsy

### Proteomic analysis by iTRAQ and NanoLC-MS/MS

**Fig. 2:**
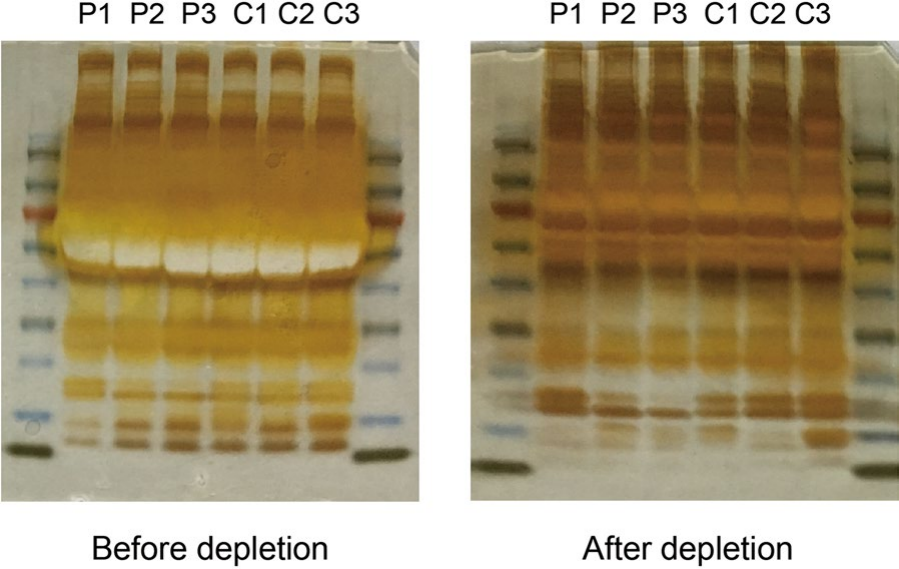
The comparison before and after depletion of high abundance protein. *P* Patient; *C* Control

Three samples from patients with LAM and healthy women were thawed to room temperature and depleted of high abundance proteins and then quantified using bicinchoninic acid assay. After dilution and denaturation, an equal amount of each sample and the protein standard were loaded on the prepared sodium dodecyl sulfate–polyacrylamide gel electrophoresis. Then, the gel was fixed, oxidized, and stained with silver nitrate in the dark till the color was developed (Fig. 2).

Briefly, an equal amount of serum proteins (100 μg) was alkylated, desalted, and trypsinized at 37℃ overnight. Resulting peptides were eluted and labeled with six different iTRAQ reagents 113–118 (AB SCIEX, Framingham, MA, USA). Then, the six pooled samples were incubated, mixed, and lyophilized. The dried samples were reconstituted and injected into Agilent high-performance liquid chromatography (HPLC) with the high pH reverse phase column (Agilent, C18, 250 mm × 4.6 mm, 5 μm). The peptides were then eluted with buffer B at a flow rate of 0.8 mL/min. A total of 16 fractions per sample were collected, lyophilized, reconstituted, and centrifuged at 12,000 g for 10 min. After resuspending, 4 μL per fraction was detected and analyzed using an Ultra 2D Plus nanoflow HPLC (Eksigent Inc., Dublin, CA, USA) via Triple TOF 6600 system (AB SCIEX, Framingham, MA, USA). Microfluidic and nanofluidic columns packed with ChromXP C18 (3 μm, 2.1 × 100 mm Eksigent) were used for trapping, desalting, and analytical separation of peptides. Labeled peptides were loaded onto the column at 2 μL/min for 15 min, followed by analytical separation at a flow rate of 300 nL/min.

The mass spectrometer data was collected in positive ion mode with a selected mass range of 350–1500 m/z. The peptides from + 2 to + 5 charge states were selected by MS/MS. The scanning method was an information-dependent acquisition mode (IDA). MS/MS spectra were acquired in the m/z range of 100–1500 using Smart information-dependent acquisition with automatic collision energy and automatic MS/MS accumulation. Detection by NanoLC-MS/MS was performed once on two biological replicates per group simultaneously.

### Bioinformatic analysis and validation

The raw peptides, protein identification, and quantification were performed using ProteinPilot v4.7 (AB SCIEX, Framingham, MA, USA) with the Paragon Algorithm against the UniProt ‘complete proteome’ human proteins database. To reduce false positive results, a minimum unused score of 1 (equivalent to 95% confidence) and a false discovery rate of less than 5% were set up to evaluate all reported proteins. We used the enrichment analysis algorithm to group the identified protein data and compared it with the database. Proteins with FoldChange values greater than 1.2 or less than 0.83 and a p-value of less than 0.05 are identified as DEPs. The heatmap and volcano plot were created by R version 4.3.9 (R Foundation for Statistical Computing, Vienna, Austria). Protein interaction diagram was created using String database. The gene ontology categories, pathway analysis, and protein–protein interaction network analysis were performed using OmicsBean (http://www.omicsbean.cn) database. Kyoto Encyclopedia of Genes and Genomes was used for pathway enrichment analysis. Moesin was selected and then validated in the enlarged serum samples from the validation cohort by Enzyme-linked immunosorbent assay (ELISA). The moesin ELISA kit was from Aviva systems biology (San Diego, CA, USA) and the validation experiments were performed according to the instruction of the manufacturer. The optical density values were measured using a microplate reader (BioTek Instruments, Inc, Winooski, VT, USA). The expression and distribution of moesin in lung tissues of healthy women and patients with LAM were detected by immunohistochemistry.

### Statistical analysis

Categorical data were presented as numbers (percentage), and continuous data were presented as mean ± standard deviation (SD) or median [interquartile range (IQR)]. Normality was evaluated in continuous variables using the Shapiro–Wilk test and the Student *t-test* or the Mann–Whitney U-test was used for comparisons between groups for continuous variables. Linear regression was used to predict the relationship between moesin and other variables. The diagnostic and therapeutic value of moesin and VEGF-D were determined using a receiver operator characteristic (ROC) curve with an area under the curve (AUC) to obtain the optimal cut-off point and corresponding sensitivity and specificity. A logistic regression model including VEGF-D and moesin was used to create a composite score. Statistical analysis was performed using Prism v9 (Graph Pad Software, San Diego, CA, USA) and SPSS version 22 (IBM SPSS Statistics, Chicago, IL, USA). The Medcalc software version 22.006 (Medcalc software, Ostend, Belgium) was used to compare the ROC curves. A *P*-value less than 0.05 was considered statistically significant.

## Results

### Baseline characteristics of the recruited cohort

A total of 84 cases with LAM were included, among which seven (8.3%) were TSC-LAM and 77 (91.7%) were S-LAM. The average age was (40.0 ± 9.6) years old. Approximately 40% had a story of pneumothorax, only seven had chylothorax and one had chylous ascites. More than 1/4 (28.6%) patients were with retroperitoneal lymphangioleiomyomas and two (2.4%) were with enlarged mediastinal lymph nodes which were confirmed to be lymphangioleiomyomas by lymph node biopsy. Chylothorax, chylous ascites, and lymphangioleiomyomas were categorized as lymphatic involvement [13], which accounted for 31.0% of the validation cohort. Renal angiomyolipomas were found in 29 (34.5%) patients, whereas liver angiomyolipomas were only present in five (6.0%). A number of 31 patients were treated with sirolimus after the baseline evaluation, one was treated with everolimus, and the remaining 52 (61.9%) patients did not receive any anti-LAM drugs. The pulmonary function tests showed a median FEV_1_/FVC of [74.6 (62.7–84.2)] %, an average FEV_1_%pred of (83.0 ± 26.7) %, and an average FVC%pred of (96.4 ± 17.7) %. The average DLCO%pred was (73.6 ± 27.0) %. The median serum level for VEGF-D was 1191.2 (644.7–2518.5) pg/mL (Table 2).

**Table 2:** Baseline clinical characteristics of patients with LAM

Variable	N = 84
Female	84 (100)
Age, y	40.0 ± 9.6
TSC-LAM	7 (8.3)
S-LAM	77 (91.7)
History of pneumothorax	35 (41.7)
History of chylothorax	7 (8.3)
History of chylous ascites	1 (1.2)
Kidney angiomyolipomas	29 (34.5)
Liver angiomyolipomas	5 (6.0)
Mediastinal LAM	2 (2.4)
Retroperitoneal LAM	24 (28.6)
Pulmonary lesions only	33 (39.3)
Extrapulmonary lesions	51 (60.7)
Lymphatic involvement	26 (31.0)
Observation	52 (61.9)
Sirolimus treatment	31 (36.9)
Everolimus treatment	1 (1.2)
Pulmonary function test	
FEV_1_, L	2.1 ± 0.7 (N = 48)
FEV_1_%pred, %	83.0 ± 26.7 (N = 48)
FVC, L	2.9 ± 0.5 (N = 48)
FVC%pred, %	96.4 ± 17.7 (N = 48)
FEV_1_/FVC, %	74.6 (62.7–84.2) (N = 48)
DLCO%pred, %	73.6 ± 27.0 (N = 45)
VEGF-D, pg/mL	1191.2 (644.7–2518.5)
Moesin, pg/mL	219.0 (118.7–260.5)

Data were presented as No. (%), mean ± SD or median (interquartile range), unless otherwise indicated

*LAM* Lymphangioleiomyomatosis or Lymphangioleiomyomas; *TSC* tuberous sclerosis complex; *VEGF-D* vascular endothelial growth factor D; *DLCO* Diffusing capacity of the lung for carbon monoxide

Patients with OCLDs averagely aged at (48.2 ± 10.9) and healthy control subjects had a median age of 37 (31–43) (Table 3). OCLDs include various definitive diagnoses such as SS, AAV, SLE, RA, etc., with SS being in the majority (29.5%). The median serum level of VEGF-D for patients with OCLDs was 367.5(248.0–492.6) pg/mL. LAM was ruled out for patients with OCLDs based on chest CT, pathological results, and other clinical data (Table 3).

**Table 3:** Baseline clinical features of patients with OCLDs and healthy control subjects

Variable	OCLDs	Healthy controls
N	44	37
Female	44 (100)	37(100)
Age, y	48.2 ± 10.9	37 (31–43)
ANCA-associated vasculitis	1(2.3)	NA
Systemic lupus erythematosus	1(2.3)	NA
Sjögren's syndrome	13(29.5)	NA
Familial vesicular disease	1(2.3)	NA
Rheumatoid arthritis	1(2.3)	NA
Unclassified connective tissue disease	2(4.5)	NA
Pathology does not support LAM	2(4.5)	NA
Qualified doctors have determined based on pulmonary CT and clinical data that LAM is not considered	23(52.3)	NA
VEGF-D, pg/mL	367.5 (248.0–492.6)	NA
Moesin, pg/mL	125.8 ± 59.9	49.6 (35.5–78.9)

Data were presented as No. (%), mean ± SD or median (interquartile range), unless otherwise indicated

*OCLDs* other cystic lung diseases; *LAM* Lymphangioleiomyomatosis; *CT* computerized tomography; *VEGF-D* Vascular endothelial growth factor D

### Proteomics and bioinformatic analysis revealed DEPs between LAM and healthy subjects

**Fig. 3:**
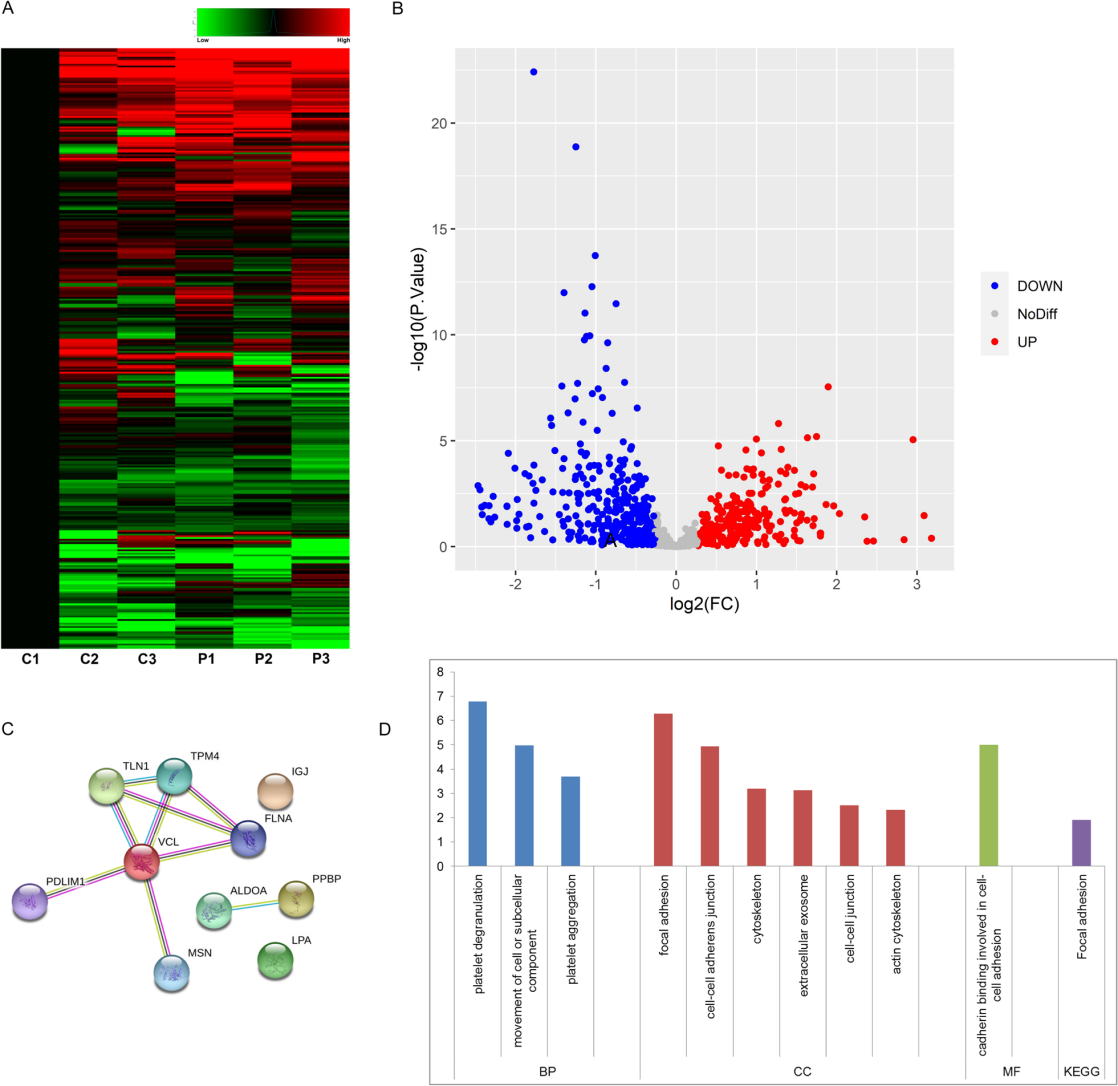
**A**, **B**, **C**, **D** Bioinformatics analysis of DEPs in serum of patients with LAM by proteomics. **A** A heat map of DEPs screened by serum proteomics; **B** A volcano plot of DEPs; **C** Interaction network of up-regulated DEPs predicted by String; **D** GO analysis in biological processes, cellular components, and molecular functions and KEGG analysis of signaling pathways of up-regulated DEPs. *P* Patient; *C* Control; *DEPs* differentially expressed proteins; *LAM* Lymphangioleiomyomatosis; *GO* Gene Oncology; *KEGG* Kyoto Encyclopedia of Genes and Genomes

Over 500 proteins were detected in the serum samples (Fig. 3A). Out of these, 31 proteins, related to lung diseases, had shown significantly differential expressions between patients with LAM and healthy woman, including 10 upregulated proteins (Fig. 3B, Table 4) and 21 downregulated proteins (Fig. 3B). The protein interaction networks and GO and KEGG pathways of upregulated DEPs were conducted (Fig. 3C, D). After conducting a literature search on the screened DEPs, we preliminarily identified moesin, which was a cytoskeleton regulatory protein that showed high expression in various tumor tissues or cells and was associated with tumor invasion or metastasis [21–23].

**Table 4:** Upregulated DEPs between LAM and healthy subjects

Accession number	Protein name	Protein abbreviation	Peptides (>95%)	Ratio: LAM versus controls	Protein function
P67936	Tropomyosin alpha-4 chain	TPM4	21	2.76	Muscle contraction regulation
P04075	Fructose-bisphosphate aldolase A	ALDOA	15	1.83	Muscle contraction regulation
O00151	PDZ and LIM domain protein 1	PDLIM1	13	2.28	Cell junction organization
P02775	Platelet basic protein	PPBP	18	3.18	Cytokine in inflammation and hematopoiesis
Q9Y490	Talin-1	TLN1	40	2.03	Integrin-mediated cell adhesion
P08519	Apolipoprotein(a)	LPA	44	4.17	Lipoprotein component
P21333	Filamin-A	FLNA	33	1.63	Actin-binding and cytoskeletal organization
P18206	Vinculin	VCL	17	1.71	Cell adhesion and cytoskeleton linkage
P01591	Immunoglobulin J chain	IGJ	54	2.10	IgM and IgA antibody polymerization
P26038	Moesin	MSN	12	1.91	Actin cytoskeleton and cell membrane linkage

*DEPs* Differentially expressed proteins; *LAM* Lymphangioleiomyomatosis

### Moesin was increased in lung tissues and serum of the patients with LAM

**Fig. 4:**
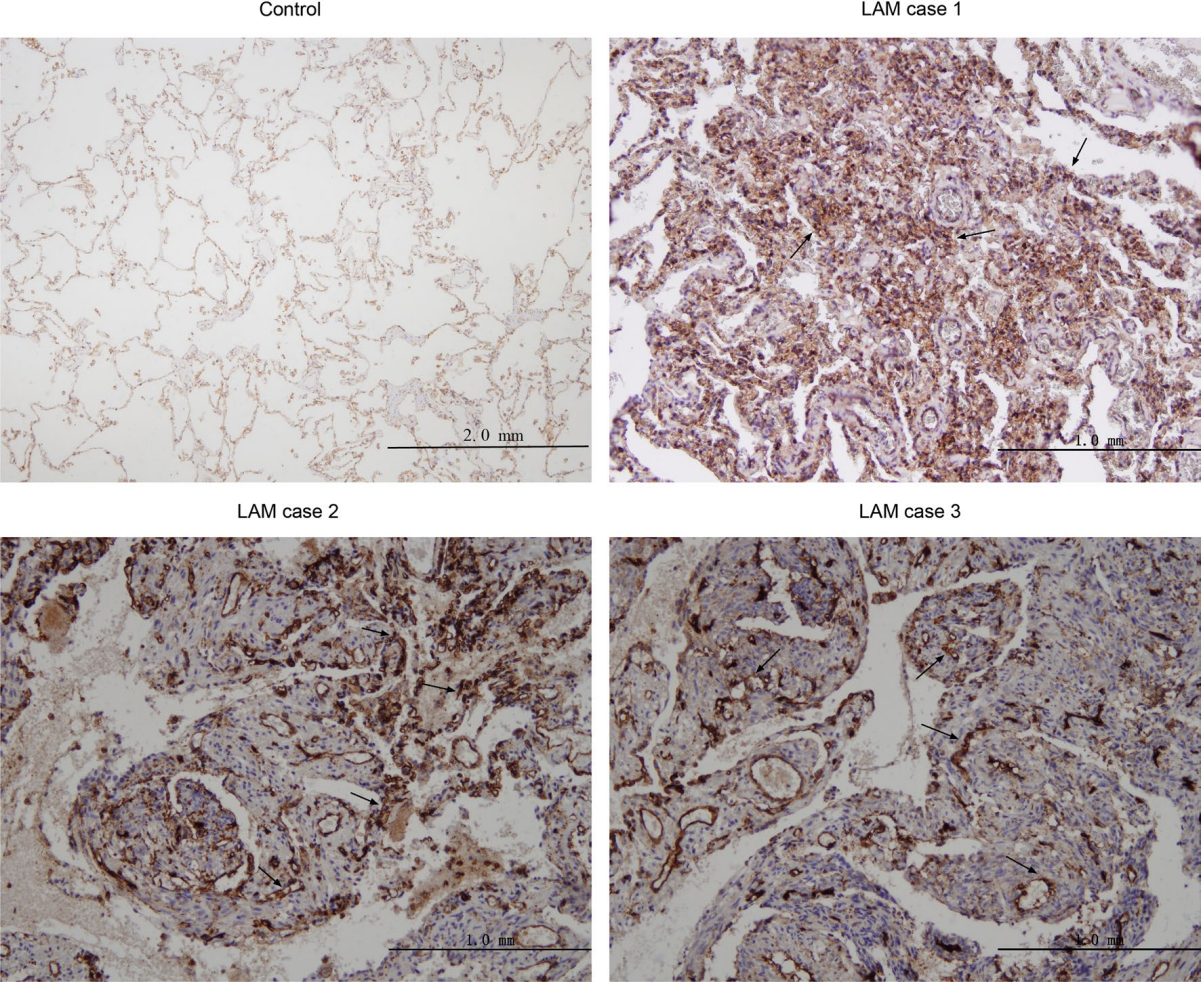
Immunohistochemical validation of moesin. *LAM* Lymphangioleiomyomatosis

Consistent with the proteomic findings, immunohistochemistry results confirmed that moesin was positively expressed in the LAM nodules as well as the surrounding microenviroment of patients with LAM, in comparison with that of healthy woman (Fig. 4).

**Fig. 5:**
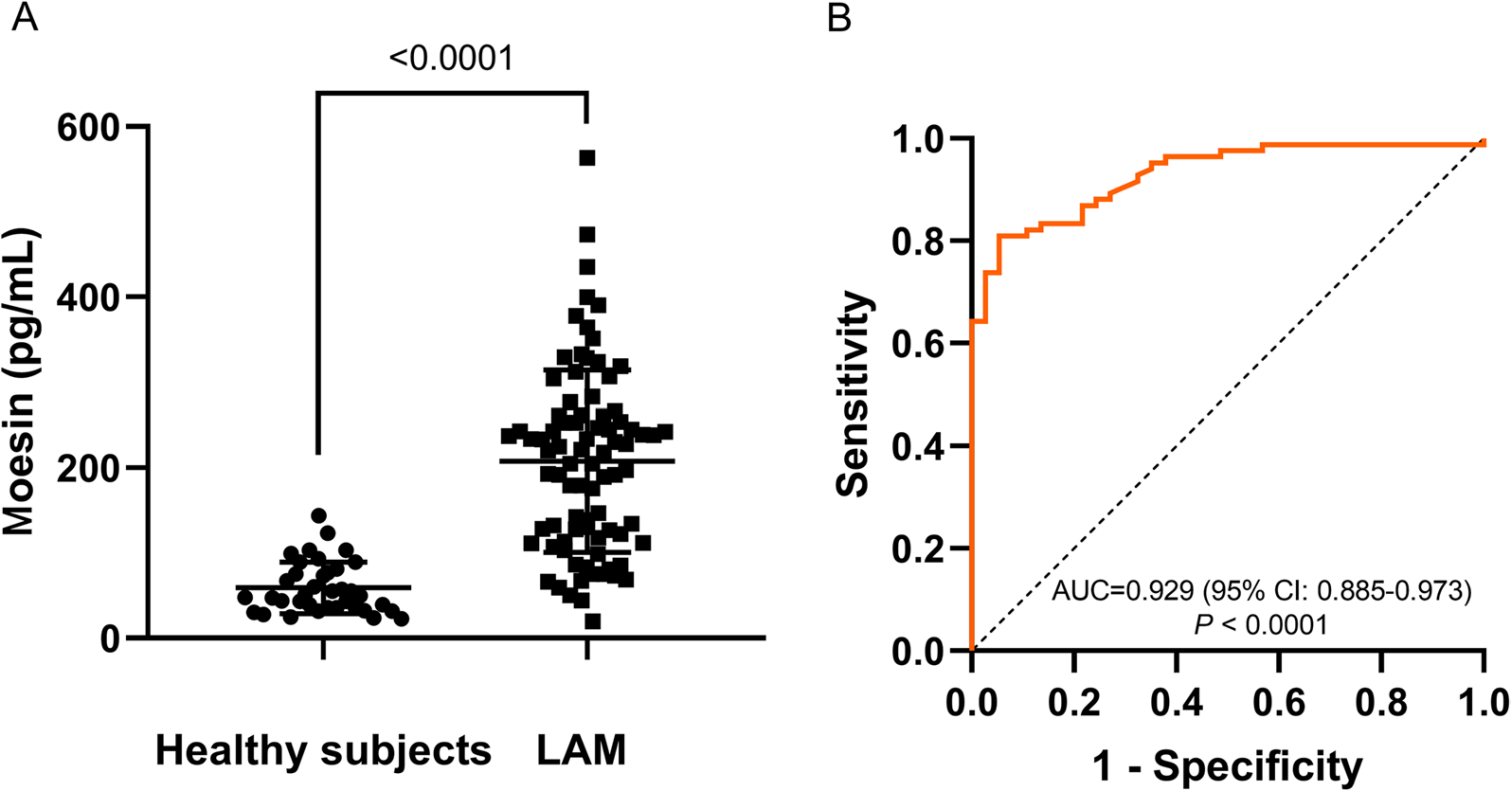
**A**, **B** Serological validation of moesin. **A** Comparison of moesin levels in serum of healthy control subjects and patients with LAM. **B** ROC curve of moesin for predicting LAM diagnosis compared to healthy control subjects. *LAM* Lymphangioleiomyomatosis; *ROC* Receiver operating characteristic; *AUC* Area under the ROC curve

**Fig. 6:**
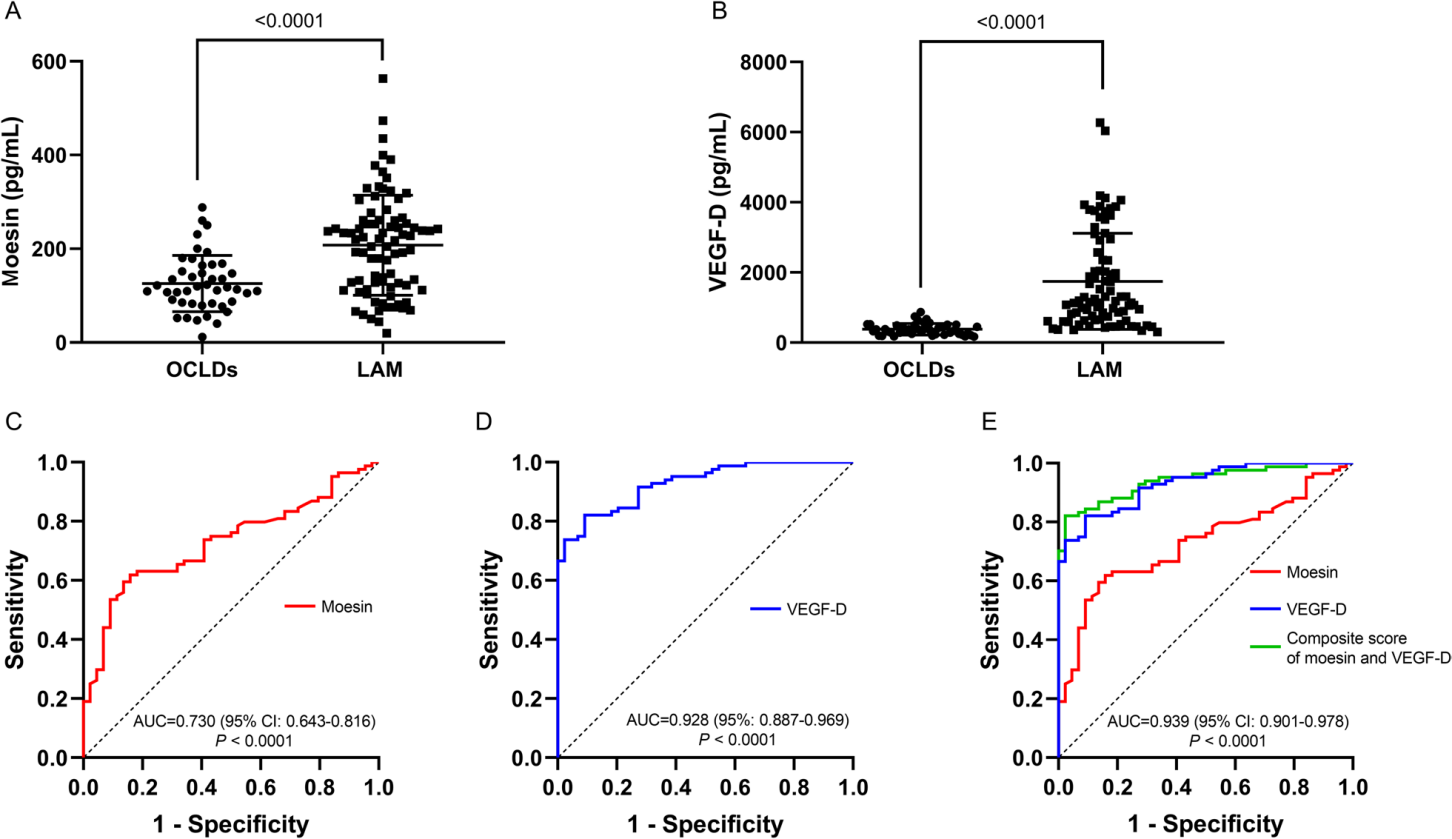
**A**, **B**, **C**, **D**, **E** Comparison of VEGF-D and moesin in patients with LAM and patients with OCLDs. **A**, Comparison of moesin in patients with LAM and patients with OCLDs. **B**, Comparison of VEGF-D in patients with LAM and patients with OCLDs. **C**, ROC curve of moesin for predicting LAM diagnosis compared to OCLDs. **D**, ROC curve of VEGF-D for predicting LAM diagnosis compared to OCLDs. **E**, ROC curve of the combination of VEGF-D and moesin for predicting LAM diagnosis compared to OCLDs. *LAM* Lymphangioleiomyomatosis; *OCLDs* other cystic lung diseases; *ROC* Receiver operating characteristic; *AUC* Area under the ROC curve; *VEGF-D* Vascular endothelial growth factor-D

The serum level of moesin in patients with LAM was significantly higher [219.0 (118.7–260.5) pg/mL] compared to healthy women [49.6 (35.5–78.9) pg/mL] (*P* < 0.0001) (Fig. 5A). In patients with LAM, both moesin and VEGF-D levels [VEGF-D: 1191.2 (644.7–2518.5) pg/mL] were significantly higher compared to those in patients with OCLDs [moesin: (125.8 ± 59.9) pg/mL, *P* < 0.0001; VEGF-D: 367.5 (248.0–492.6) pg/mL, *P* < 0.0001] (Fig. 6A, B). The moesin had an AUC of 0.929 (95% CI: 0.885–0.973) for LAM diagnosis from control subjects (Fig. 5B).

A cut-off level of 105.4 pg/mL showed sensitivity of 81.0% and specificity of 94.6%. We further studied the diagnostic value of moesin to differentiate LAM from OCLDs. Moesin had an AUC of 0.730 (95% CI 0.643–0.816, *P* < 0.0001) for differentiating LAM from OCLDs with a cut-off level of 179.0 pg/mL showing sensitivity and specificity of 61.9% and 84.1% (Fig. 6 C), while VEGF-D had an AUC of 0.928 (95% CI 0.887–0.969, *P* < 0.0001) (Fig. 6D) with a cut-off value of 589.0 pg/mL (sensitivity 82.1% and specificity 90.9%). Moreover, defining 882.1 pg/mL as a cut-off VEGF-D showed a sensitivity of 66.7% and a specificity of 100%. We further constructed a composite score by combining moesin and VEGF-D (score = − 5.252 + 0.007 × VEGF-D + 0.01 × moesin) whose AUC was 0.939 (95% CI 0.901–0.978* P* < 0.0001) (Fig. 6E). The optimal cut-off point was 0.714, with sensitivity and specificity of 82.1% and 97.7%. Likewise, a cut-off value of 0.903 showed a specificity of 100% and sensitivity of 70.2%. While moesin may not be as effective as VEGF-D alone, combining both proteins can enhance diagnostic accuracy. The composite score had a slightly preferable trend compared to VEGF-D alone, although the comparison between the AUC of VEGF-D and the composite score did not show a significant difference (*P* = 0.3426).

### Moesin was associated with lymphatic involvement in patients with LAM

**Fig. 7:**
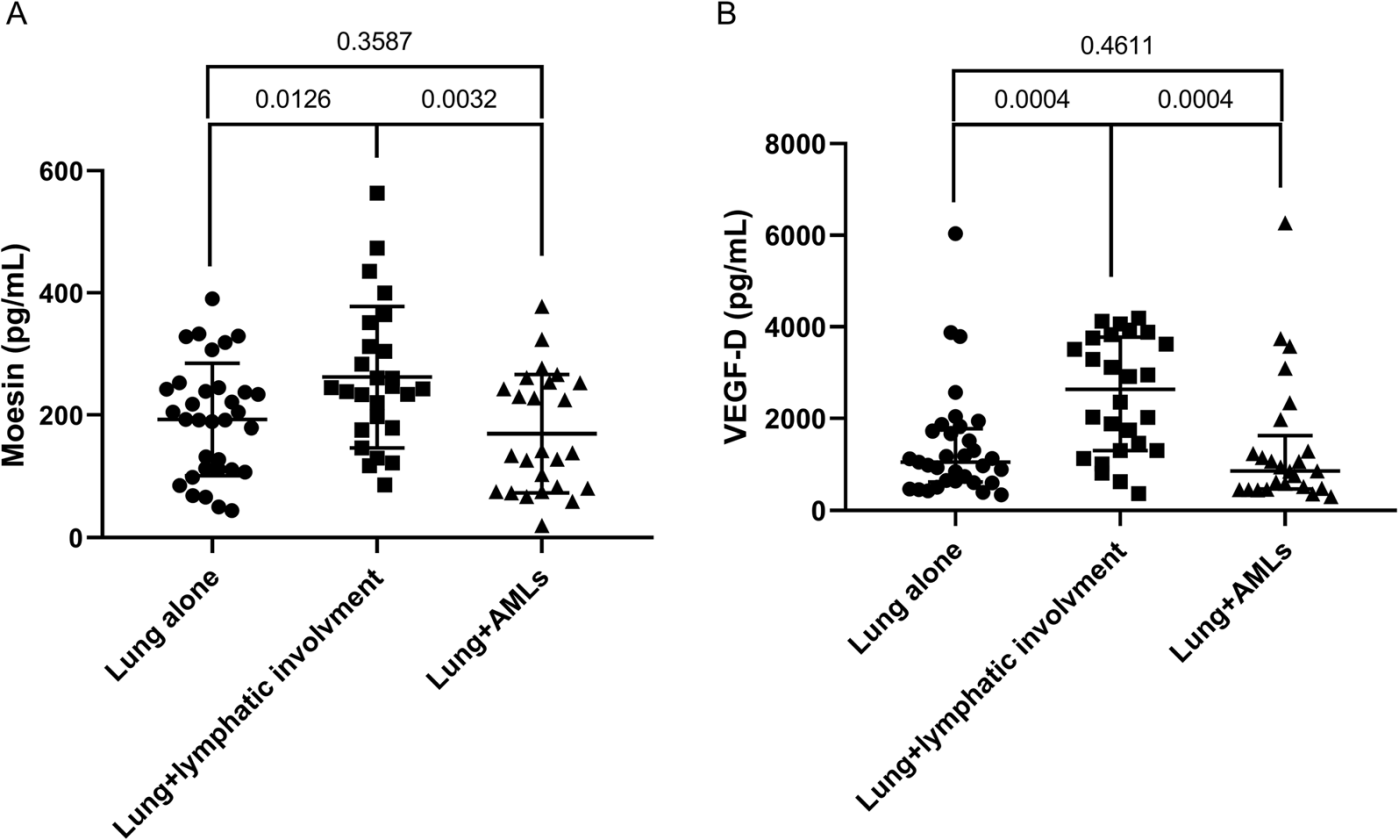
**A**, **B** Comparison of moesin and VEGF-D in different subgroups of patients with LAM. **A** Comparison of moesin among patients with lung lesions alone, patients with lymphatic involvement, and patients with lung lesions plus AMLs. **B** Comparison of VEGF-D among patients with lung lesions alone, patients with lymphatic involvement, and patients with lung lesions plus AMLs. *VEGF-D* Vascular endothelial growth factor-D; *LAM* Lymphangioleiomyomatosis; *AMLs* Angiomyolipomas

Moesin was markedly elevated in LAM patients with lymphatic involvement [(262.4 ± 115.4) pg/mL], compared with those with lung lesions alone [(192.9 ± 91.8) pg/mL, *P* = 0.0126] and lung lesion plus AMLs [(169.8 ± 96.6) pg/mL, *P* = 0.0032] (Fig. 7A). However, no significant difference in moesin was observed between patients with lung lesions plus AMLs and patients with lung lesions alone (*P* = 0.3587). Likewise, the level of VEGF-D was also higher in patients with lymphatic involvement [2635.8 (1308.9–3775.4) pg/mL] than that in those with lung lesions alone [1055.4 (626.4–1779.8) pg/mL, *P* = 0.0004] and those with lung lesions plus AMLs [865.2 (473.2–1636.8) pg/mL, *P* = 0.0004] (Fig. 7B). As well, there was no significant difference in VEGF-D between patients with AMLs and patients with lung lesions alone (*P* = 0.4611). It was implied that both moesin and VEGF-D may contribute to the lymphatic involvement in LAM.

### Moesin was associated with impaired lung function in patients with LAM

**Fig. 8:**
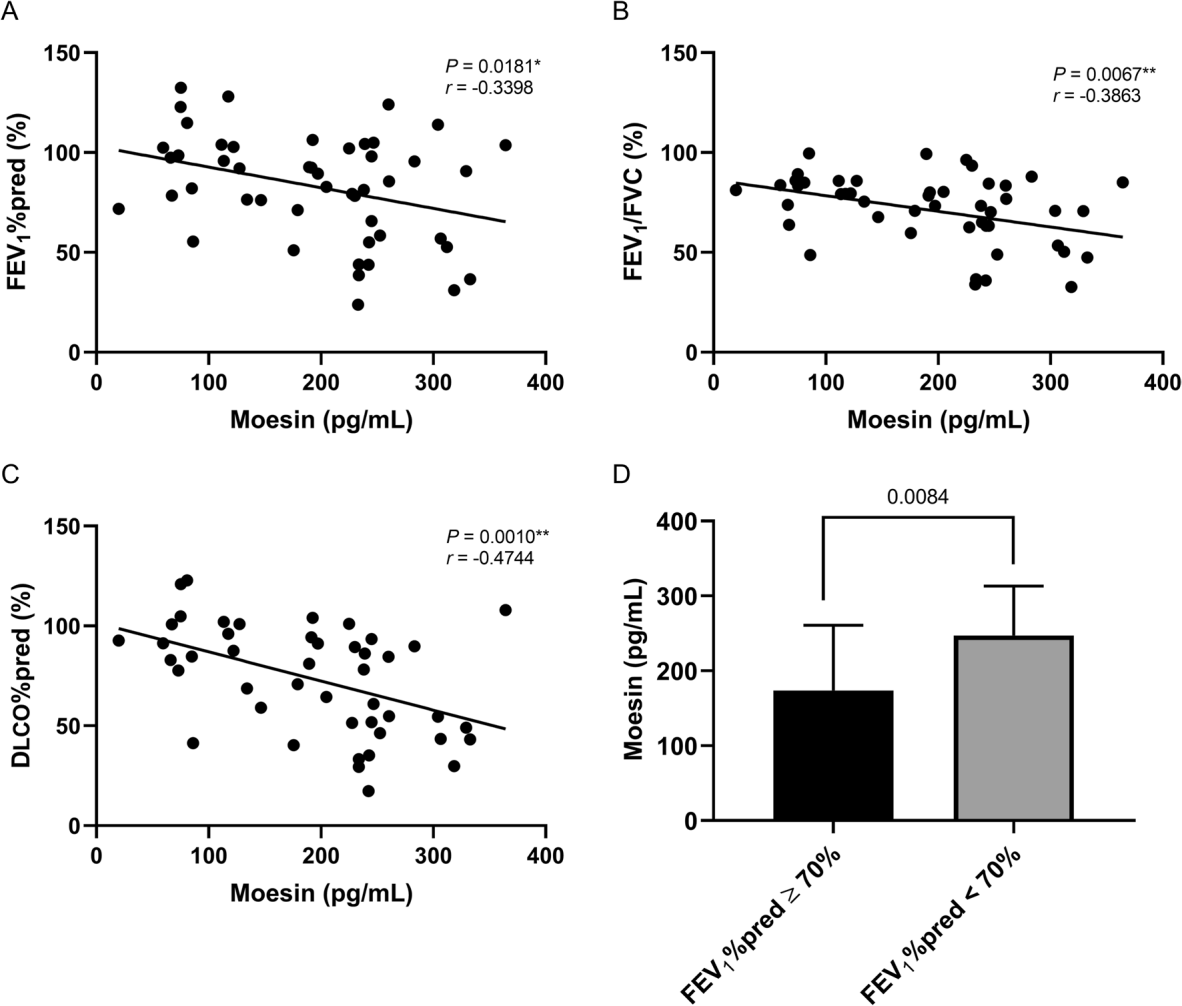
**A**, **B**, **C**, **D** The relationship between moesin and spirometry parameters. **A** The correlation between moesin and FEV1%pred. **B** The correlation between moesin and FEV1/FVC. **C** The correlation between moesin and DLCO%pred. **D** Comparison of moesin between patients with LAM with FEV1%pred < 70% and FEV1%pred ≥ 70%. *VEGF-D* Vascular endothelial growth factor-D; *LAM* Lymphangioleiomyomatosis; *DLCO* Diffusing capacity of the lung for carbon monoxide

There was no significant relationship between VEGF-D and any of spirometry parameters including FEV_1_%pred (*P* = 0.3142), FEV_1_/FVC (*P* = 0.1185), FVC%pred (*P* = 0.5348), and DLCO%pred (*P* = 0.0554), which was consistent with previous studies [14, 24, 25]. Noteworthily, moesin was negatively correlated with FEV_1_%pred (*P* = 0.0181, *r* = − 0.3398), FEV_1_/FVC (*P* = 0.0067, *r* = − 0.3863), and DLCO%pred (*P* = 0.0010, *r* = − 0.4744) (Fig. 8A, B, C), although it had no significant correlation with VEGF-D (*P* = 0.3355) or FVC%pred (*P* = 0.3808). As reported, a FEV_1_%pred less than 70% was defined as impaired lung function [19]. It was observed that higher levels of moesin were found in patients with impaired lung function [(247.3 ± 65.8) pg/mL] compared to the other patients with FEV_1_%pred of 70% and above (173.8 ± 87.1) pg/mL (*P* = 0.0084) (Fig. 8D). It suggested that moesin may act as an indispensable part involving lung function and structure damage in patients with LAM.

### Baseline moesin was elevated in patients with LAM receiving sirolimus treatment

**Fig. 9:**
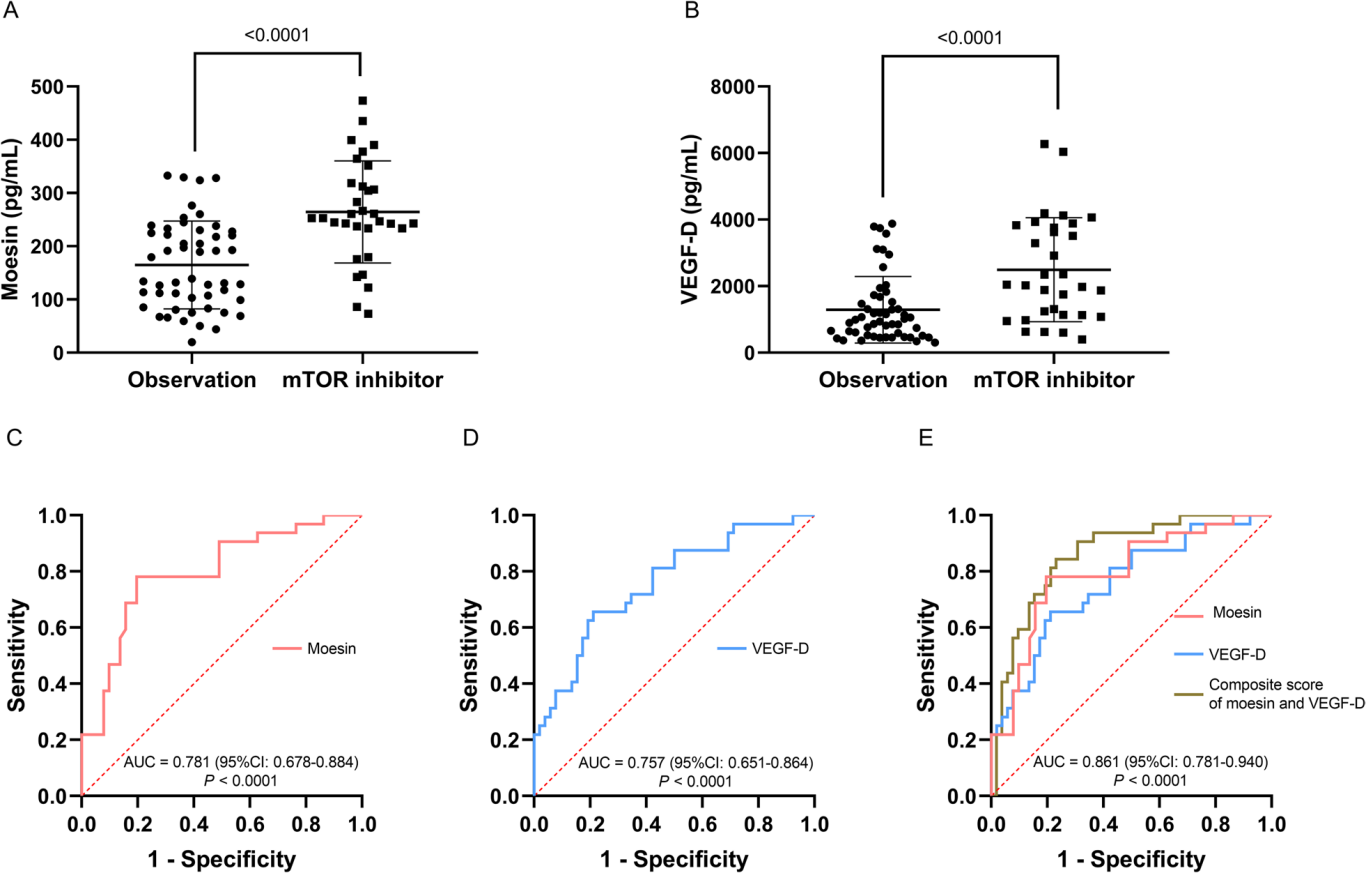
**A**, **B**, **C**, **D**, **E** Comparison of baseline levels of moesin and VEGF-D in observation group and mTOR inhibitor group. **A** Comparison of baseline moesin levels between patients in observation group and mTOR inhibitor group. **B** Comparison of baseline VEGF-D levels between patients in observation group and mTOR inhibitor group. **C** ROC curve of moesin for predicting sirolimus treatment in patients with LAM. D. ROC curve of VEGF-D for predicting sirolimus treatment in patients with LAM. **E** ROC curve of the combination of moesin and VEGF-D for predicting sirolimus treatment in patients with LAM. *VEGF-D* Vascular endothelial growth factor-D; *LAM* Lymphangioleiomyomatosis; *ROC* Receiver operating characteristic; *AUC* Area under the ROC curve

Among the 84 LAM patients in this study, 31 patients received sirolimus treatment and one received everolimus treatment, whereas the remaining patients were in the observation group. The blood samples were collected before the treatment decision. Therefore, these 32 patients were classified into the mTOR inhibitor group. The baseline moesin levels [252.6 (233.6–316.9) pg/mL] of patients receiving mTOR inhibitor treatment were significantly higher than those in the observation group [139.0 (98.9–227.7) pg/mL, *P* < 0.0001] (Fig. 9A). Similarly, VEGF-D levels in the treatment group [2035.1 (1131.8 ± 3811.8) pg/mL] were significantly higher than those in the observation group [964.8 (513.7 ± 1636.9) pg/mL, *P* < 0.0001] (Fig. 9B). Since the timing of initiating sirolimus therapy for each patient was determined according to the guidelines after complete assessment, we wondered if there was a potential biomarker that could assist in deciding when to start the sirolimus treatment. Therefore, we created ROC curves for both biomarkers to obtain the best cut-off value and the corresponding sensitivity and specificity to predict sirolimus treatment. The AUC for moesin was 0.781 (95% CI 0.678–0.884), with a best cut-off value of 233.3 pg/mL and sensitivity/specificity of 78.1%/78.9% (Fig. 9C). The AUC for VEGF-D was 0.757 (95% CI 0.651–0.864), with a best cut-off value of 1738.8 pg/mL and sensitivity/specificity of 65.6%/78.9% (Fig. 9D). We constructed a composite score integrating both biomarkers (score = − 4.334 + 0.001 × VEGF-D + 0.011 × moesin) to increase AUC to 0.861 (95% CI 0.781–0.940), which was significantly higher than that of VEGF-D alone (*P* = 0.0411) or moesin alone (*P* = 0.0581) (Fig. 9E). The best cut-off value for the composite score was 0.349, with a sensitivity/specificity of 84.4%/76.9%.

## Discussion

This study employed iTRAQ-based proteomics and bioinformatic analysis to discover the candidate protein—moesin in patients with LAM. The elevated expression of moesin was subsequently validated in serum samples from patients with LAM compared with healthy control subjects and patients with OCLDs, suggesting that moesin could emerge as a new biomarker to help differentiate LAM from OCLDs and healthy subjects. Further, serum moesin was elevated in patients with lymphatic involvement and impaired lung ventilation function and diffusion capacity, which suggested a severe condition of LAM that may link with a poor prognosis. Last but not least, patients receiving sirolimus therapy had substantially higher levels of moesin than that of the observation group. The combination of moesin with VEGF-D could provide additional clinical guidance on sirolimus treatment for LAM, which showed better efficacy than either of them individually. It is speculated that biomarkers may be taken into consideration when deciding on sirolimus treatment in the future.

Moesin is a member of the ERM family, which is primarily involved in maintaining the cytoskeleton of epithelial cells and cell motility [26]. Activation of moesin by extracellular signals causes a change in its molecular conformation, which in turn mediates the cross-linking of actin and the plasma membrane, thereby restructuring the actin cytoskeleton and regulating cell growth and migration [27]. In some malignant tumors such as gastric adenocarcinoma, breast cancer, and melanoma, upregulated moesin expression was found associated with high histological grade and advanced stage of the tumor and indicated poor prognosis [21–23]. Previously, elevated levels of moesin and ezrin were found in TSC-related cranial lesions, which co-localized with Tuberin and Hamartin in the glial cell population of the lesions, suggesting that increased moesin and ezrin may be a compensatory response following TSC gene mutation [28]. However, the role of moesin has not been explored in LAM. Our study is the first to discover the upregulated moesin expression in LAM and to explore its clinical significance.

The immunohistochemistry and serological results both demonstrated that moesin could effectively distinguish between patients with LAM and healthy volunteers. Interestingly, the immunohistochemistry results found high expression of moesin not only in LAM cells within LAM nodules but also in normal cells surrounding the lesions. It suggested that moesin may play a role in LAM by influencing the tumor microenvironment. We also found that moesin was significantly higher in LAM than that in OCLDs, although the effectiveness in distinguishing LAM from OCLDs was not as good as VEGF-D. The optimal cut-off value for VEGF-D was determined at 589.0 pg/mL. Among 15 patients with levels of VEGF-D below this threshold, eight patients exhibited levels of moesin surpassing the optimal cut-off value of 179.0 pg/mL, with two conclusively diagnosed through lung biopsy. This suggests that although the ROC curve for moesin indicates a diagnostic efficiency lower than that of VEGF-D, it may still serve as a potential complement to address the limitations of VEGF-D, especially at early stage of LAM when VEGF-D may not exhibit a significant elevation. Herein, about half of the patients with OCLDs have been diagnosed with rheumatic diseases such as SS, RA, AAV, SLE, etc., in which studies have found that moesin may serve as a novel autoantigen [29, 30]. Moesin is also involved in immune cell activation and cell polarity maintenance, possibly contributing to the pathogenesis of rheumatic diseases [31]. So, the limited diagnostic efficiency of moesin here may be attributed to its substantial increase in OCLDs patients compared to normal individuals. Indeed, the spectrum of OCLDs encompasses all of the rare cystic lung diseases such as Birt-Hogg-Dubé Syndrome and Langerhans Cell Histiocytosis, which are common differentials for LAM. We are looking forward to studying other rare cystic lung diseases in the future. When combining moesin and VEGF-D, the diagnostic efficiency was slightly increased, although without any statistical significance. Therefore, moesin may assist in diagnosing and differentiating LAM from OCLDs to some extent.

The lymphatic involvement is an important characteristic of LAM. The CT scans of patients with LAM show well-circumscribed lymphangioleiomyomas of varying sizes, appearing as lobulated masses with a fluid-filled center that may cause chylous ascites if ruptured [32]. The proliferation of LAM cells along the lymphatic vessels causes obstruction which can lead to accumulation of chylous fluid in the pleural or peritoneal spaces [33]. The lymphatic involvement is one of the extrapulmonary manifestations of patients with LAM. It was found that moesin was expressed at higher levels in patients with lymphatic involvement in patients with LAM. As is well known, invasion and metastasis are the important characteristics of LAM pathogenesis, which is defined as a malignant tumor [1]. Although LAM has been found to be caused by TSC1/2 gene mutations, the specific mechanism of metastasis is still unclear. Moesin has been shown to be involved in cell invasion and metastasis in various malignant tumors. The increased moesin levels in patients with LAM with lymphatic involvement suggested that it may be also involved in the process of LAM metastasis beyond the lungs. Moreover, AMLs are also extrapulmonary manifestations, but moesin was not elevated in patients with AMLs compared to patients with lung lesions alone, as with VEGF-D. It was suggested that moesin may affect LAM cell metastasis by disturbing the development of lymphatic vessels. In some way, this is consistent with the fact that VEGF-D plays in lymphatic endothelial cell proliferation, migration, and induction of lymphatic vessel formation [13]. However, no definitive correlation was found between moesin and VEGF-D in our studies, which indicated that moesin may participate in the pathogenesis of LAM in a different way from VEGF-D.

Moesin was correlated negatively with spirometry parameters including FEV_1_%pred, FEV_1_/FVC, and DLCO%pred, although there was no significant correlation with FVC%pred in patients with LAM. However, the range of the correlation coefficient (r) varied between 0.3398 to 0.4744, indicating that the associations between moesin and pulmonary function were of moderate strength rather than notably robust. We found that patients with higher levels of moesin may indicate impaired pulmonary ventilation and diffusion function. It is generally believed that the clustering infiltration of LAM cells around the airways leads to cell death through stimulation of matrix degradation and airway constriction, resulting in airflow obstruction and air trapping and ultimately cystic changes in the pulmonary parenchyma, manifesting as impaired lung function [33]. The exact mechanism of how moesin is involved in the process of airway obstruction in LAM remains unclear. Researchers have found that the ERM family, as cytoskeletal proteins, could regulate the permeability of pulmonary microvascular endothelial cells [34]. Other research on pulmonary endothelial cells showed that apoptosis plays an important role in the development of pulmonary emphysema [35]. Moesin may be involved in the process of cyst formation by regulating pulmonary endothelial cells, thereby influencing the lung function of patients with LAM. Further, higher moesin were found in LAM patients with significantly decreased pulmonary ventilation, indicating a need for sirolimus treatment. As well, studies have revealed that poor prognosis was associated with decreased diffusion capacity. Taveira-DaSilva et al. analyzed pulmonary function test data from 143 patients with LAM and found that DLCO was correlated with the LAM histologic score, which is a predictor of survival or time to lung transplantation [36]. The study suggested that DLCO could potentially serve as a predictor of survival or time to lung transplantation [36]. These findings suggest that moesin may serve as a serum biomarker to assess disease severity in LAM patients by reflecting their pulmonary function, especially for patients suffering from recurrent pneumothorax. To be noted, VEGF-D did not display any association with the above spirometry parameters in this study. Moesin could complement the deficiency of VEGF-D in assessing the severity of LAM.

This study found that moesin is meaningful in initiating sirolimus treatment. For patients with LAM, it is necessary to prompt evaluation of the disease severity upon diagnosis to determine whether and when sirolimus treatment is needed. Currently, there is no unified standard for treatment indications. The American Thoracic Society/Japanese Respiratory Society guidelines recommend sirolimus treatment for LAM patients with poor lung function, a rapid decline of FEV_1_, or symptomatic chylous fluid accumulations [19]. The consensus among experts in China suggested that sirolimus treatment should be also initiated promptly in patients with TSC, AMLs, or posterior peritoneal LAM [20]. In this study, we found that LAM patients receiving sirolimus treatment showed higher baseline levels of moesin and VEGF-D, which may predict the need for sirolimus treatment in patients with LAM. And combining moesin and VEGF-D improved the predictive accuracy of sirolimus treatment compared to VEGF-D alone. Therefore, moesin was not only helpful in the assessment of disease severity, but also provided additional guidance on determining the need for sirolimus treatment in LAM patients, especially for those who presented with lung lesions alone, or with pneumothorax or chyle accumulation that have difficulty in performing pulmonary function test.

This study has several limitations. The discovery cohort included only three cases and three controls, which may limit our insight into all aspects of LAM. To make up for this, we expanded the validation cohort. Due to ethical considerations, patients undergoing pulmonary function tests exhibited milder pulmonary function impairment, potentially affecting the representativeness of the data. The relationship between moesin and other important information in LAM patients including the results of CT grades, arterial blood gas analysis, six-minute walk distance, and St. George's Respiratory Questionnaire should be studied further. As a cross-sectional study, it lacks follow-up data to study how moesin changes as the disease develops or treatment continues. Additionally, in vivo and vitro experiments are needed to verify the possible role of moesin in the pathogenesis of LAM.

## Conclusion

This study utilized a proteomic and bioinformatic analysis to identify moesin as a novel biomarker for LAM. Moesin emerged as a promising biomarker improving diagnosis and assessment of LAM. The elevated levels of moesin in serum not only implied the involvement of lymphatics but also served as an indicator of impaired lung function in patients with LAM, indicating a more advanced stage. Moesin, especially when combined with VEGF-D, exhibited its potential in assessing disease severity and making therapeutic decisions for LAM.

## Data Availability

All data generated or analyzed during this study are included in this published article and its Additional files.

## Abbreviations

LAM: Lymphangioleiomyomatosis
VEGF-D: Vascular endothelial growth factor D
iTRAQ: Isobaric tags for relative and absolute quantitation
OCLDs: Other cystic lung diseases
ELISA: Enzyme-linked immunosorbent assay
AMLs: Angiomyolipomas
TSC: Tuberous sclerosis complex
TSC-LAM: Tuberous sclerosis complex associated lymphangioleiomyomatosis
S-LAM: Sporadic lymphangioleiomyomatosis
mTORC1: Mammalian target of rapamycin complex 1
SMA: Smooth muscle antibodies
HMB-45: Human melanoma black-45
HRCT: High-resolution computed tomography
LC–MS/MS: Liquid chromatography-mass spectrometry
MS: Mass spectrometry
DEPs: Differentially expressed proteins
ERM: Ezrin-Radixin-Moesin
SS: Sjögren's syndrome
AAV: ANCA-associated vasculitis
SLE: Systemic lupus erythematosus
HPLC: High-performance liquid chromatography
SD: Standard deviation
IQR: Interquartile range
ROC: Receiver operator characteristic
AUC: Area under the curve
